# Gender differences in response to acute and chronic angiotensin II infusion: a translational approach

**DOI:** 10.14814/phy2.12434

**Published:** 2015-07-06

**Authors:** Tsjitske J Toering, Anne Marijn van der Graaf, Folkert W Visser, Hendrik Buikema, Gerjan Navis, Marijke M Faas, A Titia Lely

**Affiliations:** 1 Division of Nephrology, Department of Internal Medicine, University of Groningen, University Medical Center GroningenGroningen, The Netherlands; 2 Division of Medical Biology, Department of Pathology and Medical Biology, University of Groningen, University Medical Center GroningenGroningen, The Netherlands; 3 Department of Clinical Pharmacology, University of Groningen, University Medical Center GroningenGroningen, The Netherlands; 4 Department of Obstetrics & Gynaecology, University of Groningen, University Medical Center GroningenGroningen, The Netherlands

**Keywords:** Angiotensin II, proteinuria, renal damage, renal hemodynamics, sex

## Abstract

Women with renal disease progress at a slower rate to end stage renal disease than men. As angiotensin II has both hemodynamic and direct renal effects, we hypothesized that the female protection may result from gender differences in responses to angiotensin II. Therefore, we studied gender differences in response to angiotensin II, during acute (human) and chronic (rats) angiotensin II administration. In young healthy men (*n* = 18) and women (*n* = 18) we studied the responses of renal hemodynamics (^125^I-iothalamate and ^131^I-Hippuran) and blood pressure to graded angiotensin II infusion (0.3, 1.0, and 3.0 ng/kg/min for 1 h). Men had increased responses of diastolic blood pressure (*P* = 0.01), mean arterial pressure (*P* = 0.05), and a more pronounced decrease in effective renal plasma flow (*P* = 0.009) than women. We measured the changes in proteinuria and blood pressure in response to chronic administration (200 ng/kg/min for 3 weeks) of angiotensin II in rats. Male rats had an increased response of proteinuria compared with females (GEE analysis, *P* = 0.001). Male, but not female, angiotensin II-treated rats had increased numbers of renal interstitial macrophages compared to sham-treated rats (*P* < 0.001). In conclusion, gender differences are present in the response to acute and chronic infusion of angiotensin II. Difference in angiotensin II sensitivity could play a role in gender differences in progression of renal disease.

## Introduction

Women generally have a lower risk for developing cardiovascular disease (CVD) and chronic kidney disease (CKD) than men. They also progress slower to end stage renal disease (ESRD) after a renal insult (Silbiger and Neugarten 1995). The renin-angiotensin aldosterone system (RAAS), with angiotensin II (ang II) as a principal effector, is an important mediator of renal and cardiovascular physiology and pathophysiology with hemodynamic, pro-inflammatory, and pro-fibrotic effects. Thus, differences in responsiveness to ang II might provide a possible explanation for the gender difference in progression to renal disease. Indeed, in the last few years gender differences in the components and regulation of the RAAS have been recognized to be of functional importance (Sullivan 2008; Hilliard et al. 2013).

Elucidation of gender differences is important, since this may translate into gender-specific treatment and subsequently to better outcomes in men and women. The importance of such studies is supported by the evidence suggesting gender differences in the efficacy and effects of anti-hypertensive medication in general and more specifically in RAAS blockade (Ruggenenti et al. 2000; Falconnet et al. 2004; Franconi et al. 2007; Hudson et al. 2007). In cardiovascular studies, women appear to be less responsive to angiotensin-converting enzyme inhibition (ACE-i) than men (Falconnet et al. 2004; Hudson et al. 2007), while proteinuric women responded better to treatment with ACE-i than men (Ruggenenti et al. 2000). Men require larger doses of ang II type 1 receptor (AT_1_-R) blocker to achieve the same blood pressuring lowering effect as women (Miller et al. 2006). These results illustrate the importance of a better understanding of the effect of gender differences in the RAAS.

Human gender differences in RAAS function have also been observed in responsiveness to ang II infusion. Two studies in healthy normotensive men and women (Gandhi et al. 1998; Miller et al. 1999) showed differences in the renal responsiveness to graded ang II infusion, whereas blood pressure responses were similar, pointing toward specific renal differences between men and women. Both studies, however, did not study women at a fixed time-point of the menstrual period. It is well known that this may be of great influence on both the RAAS and renal hemodynamics. Indeed, Miller et al. showed that baseline characteristics for blood pressure, effective renal plasma flow (ERPF), renal blood flow (RBF), filtration fraction (FF), and renal vascular resistance (RVR) are affected by 17ß-estradiol concentrations (Miller et al. 1999). Moreover, they also showed that 17ß-estradiol concentrations affected the response of ERPF and aldosterone to ang II infusion (Miller et al. 1999). Therefore, it is important to standardize the time-point of the menstrual cycle in small physiological studies.

To substantiate, however, the relevance of differences in the RAAS for the susceptibility to chronic renal damage, it is crucial to demonstrate differences in responsiveness to chronic ang II administration as well. This is cumbersome to study in humans. Available animal data on ang II infusion so far also show increased responsiveness to angiotensin in males compared with females (Sampson et al. 2008; Tatchum-Talom et al. 2005; Xue et al., 2005; Ebrahimian et al. 2007). Whether the effects of chronic ang II infusion on proteinuria are also sex dependent has not been studied.

Therefore, in the present translational study we combined human and animal experiments to investigate gender differences in the response to ang II. In healthy young men and women (during mid-follicular phase) on standardized sodium intake, we studied the responses of blood pressure and renal hemodynamics to an acute graded ang II infusion. The effects of ang II on blood pressure, proteinuria, and morphological markers of proinflammatory pathways and fibrosis in the kidney were studied in healthy young male and female rats after 3 weeks of chronic ang II infusion.

## Materials and Methods

### Human experiments

#### Study population

The study population was recruited from the ongoing Groningen Renal Hemodynamic Cohorts (GRECO) program. The GRECO program hosts and harmonizes studies on renal hemodynamics at the renal function unit of the University Medical Center Groningen, by standardization of methodology, including harmonization of study protocols and calibration over time. This allows combined analyses in subjects from different substudies. The current study population consisted of 36 healthy, Caucasian subjects (women, *n* = 18; men, *n* = 18). Women were studied in the RETAP project, and compared with men from the Gene-Environment project (Lely et al. 2010; Visser et al. 2008a,b). For the parameters used in the present study, the protocols were prospectively designed to be identical in design and measurements. All subjects were nonsmokers and normotensive, had a sitting systolic blood pressure < 140 mmHg and diastolic blood pressure < 90 mmHg measured by an automatic sphygmomanometer (Dinamap®, GE Medical Systems, Milwaukee, WI) and were not treated with antihypertensive medication. Their medical history revealed no significant diseases. Subjects with obesity (BMI>30 kg/m^2^ at screening) were excluded. Physical examination and electrocardiography did not reveal any abnormalities. None of the women were users of oral contraceptive medication. Both studies were approved by the local medical ethical committee (METc number: RETAP study 2010/294, Gene-Environment study 2001/012), and all subjects gave written informed consent in accordance with the Declaration of Helsinki. The RETAP study was registered in the Netherlands National Trial Register (www.trialregister.nl; trial registration number: 2635) as REsponse To Angiotensin II in formerly Preeclamptic women (RETAP) study.

#### Study protocol

The study protocol consisted of a 7-day period on a standardized sodium diet (aim: 200 mmol Na^+^/day). For assessment of dietary compliance and the achievement of a stable sodium balance, 24 h-urine was collected at day 3 and day 6. As both renal hemodynamics and ang II responsiveness are greatly influenced by female sex hormones (Pechere-Bertschi and Burnier 2004), all measurements in women were performed during the mid-follicular phase (day 7 ± 2 of menstrual cycle). At day 7 of the study protocol, the subjects reported at the research unit at 8 am after an overnight fast. Body weight, length and waist-to-hip ratio were measured at the start of this day. Intravenous cannulas were inserted into both forearms, one for drawing blood samples, the other for infusion of ang II. Subjects received standardized meals and fluids during the day, with sodium intake adjusted to the prescribed diet. To ensure sufficient urine output, infusion of 250 mL/h of 5% glucose was administered and every hour 250 mL of oral fluids were provided.

Glomerular filtration rate (GFR) and ERPF were measured from the clearance of constantly infused radio-labeled tracers, ^125^I-iothalamate and ^131^I-Hippuran, respectively, in semi-supine position in a quiet room. After drawing a blank blood sample, a priming solution, containing 0.04 mL/kg body weight (0.04 MBq of ^125^I-iothalamate and 0.03 MBq of ^131^I-Hippuran per milliliter saline) plus an extra bolus of 0.06 MBq of ^125^I-iothalamate was given, followed by constant infusion at 12 mL/h of the same solutions. After a 2 h stabilization period, stable plasma concentrations of both tracers were attained, and the clearance periods started. The clearances were calculated using the formula U*V/P for GFR and I*V/P for ERPF. U*V represents the urinary excretion of the tracer, I*V represents the infusion rate of the tracer, and P represents the tracer value in plasma at the end of each clearance period. The urinary clearance of ^125^I-iothalamate was multiplied by the ratio of plasma-to-urinary clearance of ^131^I-hippuran to correct for voiding errors (Apperloo et al. 1996; Visser et al. 2008a,b). GFR and ERPF were indexed for body surface area (BSA), by dividing the raw sample by BSA and multiplying it with 1.73 m^2^. BSA was calculated according to the DuBois-DuBois formula (Du Bois and Du Bois 1989).

Blood pressure and heart rate were measured by using an automated sphygmomanometer (Dinamap®; GE Medical Systems). The appropriate blood pressure cuff was determined on the basis of arm circumference. Mean arterial pressure (MAP) was calculated as diastolic pressure plus one-third of the pulse pressure.

Baseline values for blood pressure, GFR and ERPF were obtained from 10 am to 12 pm at 15 min intervals. Between 12 pm and 3 pm ang II (Clinalfa, Merck Biosciences AG, Läufelfingen, Switzerland) was administered intravenously at a constant rate in doses of 0.3, 1 and 3 ng/kg/min each during 1 h. During these ang II infusions blood pressure was measured at 5-min intervals.

#### Urine sampling and analysis

Urine samples were drawn from the 24 h-urine collected by all subjects. The levels of urinary sodium, potassium, and urea were assessed by an automated clinical chemistry analyzer (Roche Modular, Basel, Switzerland).

### Animal experiments

#### Experimental set up

Experiments were conducted under protocols approved by the Animal Ethical Committee of the University of Groningen. Four-month-old female (*n* = 25) and male (*n* = 19) Wistar rats (Harlan Inc, Horst, the Netherlands) were kept in a 12-h light–dark cycle and constant room temperature, with food and water freely available in the home cages. Both female and male rats were divided in two groups: one sham-pump-treated group (control; female, *n* = 14; male, *n* = 10), and one group receiving ang II infusion continuously via an osmotic minipump (female, *n* = 11; male, *n* = 9). The infusion rates of ang II infusion were similar between males and females, that is, we infused ang II at a rate of 200 *μ*g/min/kg (ang II in saline containing 0.01 N acetic acid). For minipump implantation rats were anesthetized with isoflurane and oxygen and minipumps were implanted intraperitoneally (Alzet, Cupertino, CA, model 2004). In all rats, blood pressure and proteinuria were measured at baseline (day -1, i.e. before minipump implantation) and at weekly intervals after pump implantation (days 7, 14, and 21). Subsequently, after 3 weeks of infusion (day 21) rats were sacrificed and the left kidney was collected. Parts of the kidney were harvested and fixed in 4% paraformaldehyde in PBS or snap frozen.

#### Measurement of blood pressure

Blood pressure was measured using an indirect tail-cuff plethysmographic method with a blood pressure monitor (Apollo 179; IITC Life Science, Woodland Hills, CA). All rats were conscious during the measurements. In order to reduce spontaneous variation in blood pressure, rats were extensively trained for a period of 4 weeks on a daily basis to get used to the tail-cuff method. Prior to blood pressure readings, rats were optimally warmed using a warmth lamp to induce vasodilation of the tail vein. Readings were repeated 10 times and after excluding the lowest value, the average of the lowest three values for systolic blood pressure were used for further analysis.

#### Blood and urine sampling and analysis

Twenty-four-hour urine samples were collected from all rats. To this purpose, rats were placed in metabolic cages on days -1, 7, 14, and 21. Urinary concentration of protein (Pyrogallol Red – Molybdate Complex) was determined as previously described (Watanabe et al. 1986) and 24-h excretion rates were calculated. A blood sample was taken at sacrifice in a precooled EDTA tube (day 21) and immediately centrifuged at 4°C, 956 g for 10 min. Plasma was subsequently stored at -80°C until analysis. Urinary and plasma creatinine concentrations were determined (CREA plus, cobas, Roche Modular) from samples collected on day 21 and creatinine clearance was calculated according to the standard formula ((urinary creatinine (mmol)*1000)/plasma creatinine (*μ*mol)*(urine volume/1440)).

#### Immunohistochemistry

After PFA fixation of renal tissue for 24 h, and 70% alcohol for at least 24 h, tissue was processed for paraffin embedding according to standard methods. For immunohistochemistry, 2 *μ*m sections were cut. Total macrophages/monocytes (ED-1; 1:100 diluted, AbD Serotec, Düsseldorf, Germany) and CD206-positive macrophages (representing type 2 macrophages; 1:1000 diluted; Abcam, Cambridge, UK) were stained as previously described (Melgert et al. 2012). Numbers of interstitial ED-1 and CD206-positive cells were determined by manually analyzing 30 randomly selected cortical fields per kidney (40× magnification), excluding fields with glomeruli. For each cortical field, the number of positive cells was counted.

The prefibrotic marker for myofibroblast transformation, alpha-smooth muscle actin (*α*-SMA), was detected using a murine monoclonal antibody (*α*-SMA; clone 1A4; Sigma) as previously described (Rook et al. 2005). Sections stained for *α*-SMA were scanned using an Aperio ScanScope CS and analyzed with Aperio ImageScope v10.2.2.2319 (Aperio, Vista, CA). The “Positive pixel Count V9” algorithm was used to analyze *α*-SMA-positive pixels in 30 randomly selected cortical fields per kidney after excluding vessels and glomeruli. The positive surface area (number of positive pixels) was divided by the total area of the field measured, providing a number of *α*-SMA-positive pixels corrected for the area analyzed. Researchers were blinded for group allocation of the rats while analyzing the kidney slides.

#### Gene expression analysis

Total kidney RNA from homogenized renal cortex was isolated with TRIzol Reagent (Invitrogen) following the manufacturer’s instructions. Total RNA was quantified using a NanoDrop ND1000 spectrophotometer (NanoDrop Technologies Inc., Wilmington, DE). cDNA synthesis was performed as described before (Plosch et al. 2010). Real-time RT-PCR was performed using Lightcycler 480 (Roche, Applied Science, Penzberg, Germany) and Applied Biosystems reagents according to the manufacturer’s instructions. Expression levels were normalized to those of 18S ribosomal RNA, which was analyzed in separate runs. Primers and probes for the angiotensin II type 1 (AT_1_-R) and the angiotensin II type 2 (AT_2_-R) receptors were obtained from Applied Biosystems (TaqMan Gene Expression Assays, AT_1_-R: Rn00578456_m1 and AT_2_-R: Rn00560677_m1). The sequence for 18S (M11188) (sense primer, antisense primer, and probe, respectively; all from 5′ to 3′) was: CGGCTACCACATCCAAGGA, CCAATTACAGGGCCTCGAAA, CGCGCAAATTACCCACTCCCGA.

### Data analysis and power analysis

Statistical analysis was performed using spss for Windows (Version 20.0; IBM SPSS, New York, NY). Parametric data are presented as mean ± standard deviation (SD) or mean ± standard error of the mean (SEM) in text, table, and figures and analyzed using Student *t*-test. Nonparametric data are presented as median (25th–75th percentile) and analyzed using Mann–Whitney *U*-test or Kruskal–Wallis test.

The responses to ang II infusion in humans (blood pressure and renal hemodynamics) and rats (blood pressure and proteinuria) were analyzed by generalized estimating equations (GEE) analysis. Variables with a skewed distribution were log transformed to fulfill the criteria for GEE analysis. Statistical significance was accepted at *P* ≤ 0.05.

## Results

### Human study

#### Baseline characteristics

Baseline characteristics of the men and women are shown in Table1. Age was not different between the groups. In men body height and body weight were significantly higher. BMI and waist-to-hip ratio were not different between the groups. No significant differences were found in urinary sodium, potassium, or urea excretion between the groups, which reflect equal sodium, potassium, and protein intake in men and women during the week of standardized diet. At baseline, men had a significantly higher systolic blood pressure than women, but diastolic blood pressure and MAP were similar in men and women. Men had a significantly lower heart rate. With regard to renal hemodynamics, at baseline no differences in GFR were found between the groups, but men had a significantly higher ERPF than women.

**Table 1:** Baseline characteristics of human subjects

	Female (*n* = 18)	Male (*n* = 18)	*P*
Age, years	36 ± 5	31 ± 11	0.092
Height, m	171 ± 5	184 ± 6	**<0.001**
Body weight, kg	68.0 ± 8.4	78.7 ± 8.0	**<0.001**
BMI, kg/m^2^	23.2 ± 2.7	23.2 ± 2.2	0.969
Waist/Hip ratio	0.83 ± 0.04	0.85 ± 0.08	0.397
Urinary sodium, mmol/24 h	221 ± 63	200 ± 69	0.356
Urinary potassium, mmol/24 h	80 ± 34	68 ± 22	0.267
Urinary urea, mmol/24 h	339 ± 89	383 ± 82	0.132
Systolic blood pressure, mmHg	115 ± 8	124 ± 12	**0.012**
Diastolic blood pressure, mmHg	71 ± 8	73 ± 8	0.403
Mean arterial pressure, mmHg	85 ± 8	90 ± 8	0.098
Heart rate, p/min	67 ± 8	57 ± 8	**<0.001**
GFR, mL/min/1.73 m^2^	109 ± 15	111 ± 14	0.693
ERPF, mL/min/1.73 m^2^	380 ± 69	493 ± 78	**<0.001**

BMI, body mass index; GFR, glomerular filtration rate; ERPF, effective renal plasma flow.

Data are expressed as mean ± SD. *P*-value < 0.05 is statistically significant (bold).

#### Blood pressure response to acute ang II infusion in men and women

**Figure 1:**
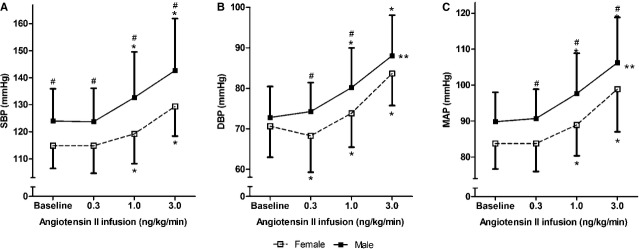
Mean (± SD) systolic blood pressure, diastolic blood pressure and mean arterial pressure during acute angiotensin II infusion in females (open squares) and males (closed squares). Abbreviations: SBP, systolic blood pressure; DBP, diastolic blood pressure; MAP, mean arterial pressure. *Significantly different from baseline (Student’s *t*-test), *P* ≤ 0.05. ^#^Significantly different from females after infusion of the same dose of angiotensin II (student *t*-test), *P* ≤ 0.05. **Curves of males and females significantly different (GEE analysis), *P* ≤ 0.05. After correction for baseline value, the gender difference in MAP responses became borderline significant (*P* = 0.08), gender difference in diastolic blood pressure response to ang II remained statistically significant (*P* = 0.01).

The responses of blood pressure to increasing doses of ang II infusion in men and women are shown in Figure1. It shows that the lowest dose, 0.3 ng/kg/min was a nonpressor dose, with even a significant decrease in diastolic blood pressure compared to baseline in women. Significant dose-dependent increases occurred during 1.0 and 3.0 ng/kg/min in both groups. During several infusion steps, men had higher systolic blood pressure (baseline, 0.3, 1.0 and 3.0 ng/kg/min), diastolic blood pressure (0.3 and 1.0 ng/kg/min) and MAP (0.3, 1.0 and 3.0 ng/kg/min) than women. Analyzing the dose response curve as a whole by GEE analysis indicated that the responses, albeit small, were significantly different for diastolic blood pressure (*P* = 0.01) and MAP (*P* = 0.05). After correction for baseline value, the gender difference in MAP responses became borderline significant (*P* = 0.08), but the gender difference in diastolic blood pressure response to ang II remained statistically significant (*P* = 0.01).

#### Renal hemodynamic response to acute ang II infusion in men and women

**Figure 2:**
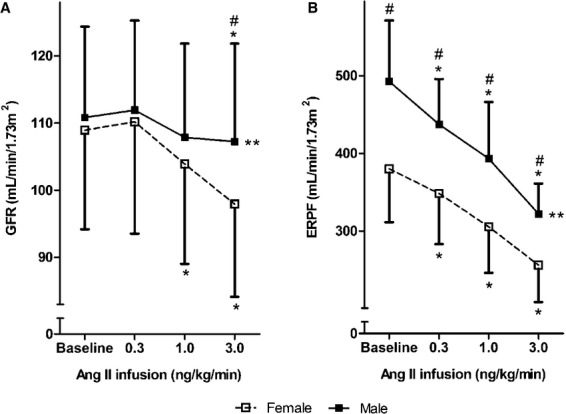
Mean (± SD) glomerular filtration rate (A) and effective renal plasma flow (B) during acute angiotensin II infusion in females (open squares) and males (closed squares). Abbreviations: GFR, glomerular filtration rate; ERPF, effective renal plasma flow; ang II, angiotensin II.*Significantly different from baseline (Student’s *t*-test), *P* ≤ 0.05. ^#^Significantly different from females after infusion of the same dose of angiotensin II (student t-test), *P* ≤ 0.05. ** curves of males and females significantly different (GEE analysis), *P* ≤ 0.05.

Figure2 shows the responses of GFR (panel A) and ERPF (panel B) during ang II infusion in men and women. Women responded to ang II infusion by a significant decline in GFR during the 1.0 ng/kg/min and 3.0 ng/kg/min infusion rate, while men responded to ang II by maintaining GFR at all infusion doses. At the dose of 3.0 ng/kg/min GFR was significantly lower in women than in men (*P* = 0.001). Analyzing the dose–response curves as a whole by GEE analysis indicated that the response was significantly different between men and women for GFR (*P* = 0.01; corrected for baseline values).

ERPF significantly and progressively decreased during increasing doses of ang II in men and women. Analyzing the dose–response curve as a whole by GEE analysis indicated that the response was significantly different between men and women for ERPF (*P* = 0.009; corrected for baseline value).

### Animal study

The baseline body weight was significantly higher in male rats compared to female rats, while ang II treatment did not affect body weight (males: sham-treated rats 405 ± 18 g, ang II-treated rats 397 ± 9 g; females: sham-treated rats 268 ± 18 g, ang II-treated rats 271 ± 18 g; *P* < 0.001).

#### Responses of blood pressure and urinary protein excretion to chronic ang II infusion

**Figure 3:**
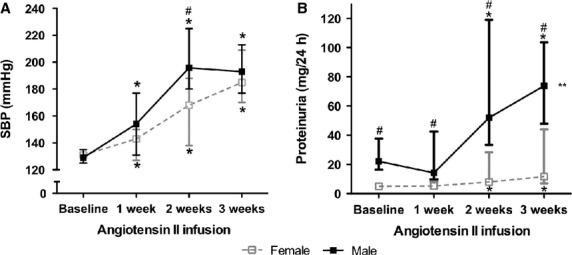
Median (with 25th–75th percentile) systolic blood pressure (A) and proteinuria (B) during chronic angiotensin II infusion in female (open squares) and male (black squares) rats; *Significantly different from baseline (Mann–Whitney *U*-test), *P* ≤ 0.05. ^#^Significantly different from females (Mann–Whitney *U*-test), *P* ≤ 0.05. **Curves of males and females significantly different (GEE analysis: interaction between angiotensin II proteinuria response and sex).

At baseline, blood pressure did not significantly differ between both groups. However, at baseline, male rats had a significantly higher 24 h urinary protein excretion than female rats (Fig.3). In sham rats, systolic blood pressure did not change over the 3-week treatment period in either group (results not shown). After chronic ang II infusion both male and female rats had a significant increase in systolic blood pressure (Fig.3). Male rats showed a more rapid increase in systolic blood pressure than female rats, reaching a plateau after 2 weeks of ang II infusion. At that time-point, male rats had a significantly higher systolic blood pressure than female rats.

In the sham-treated rats no significant changes in proteinuria were observed during the 3 weeks of treatment (results not shown). During 3 weeks of ang II infusion, which was similar in males and females, both male and female rats showed a significant increase in proteinuria, starting after the first week (Fig.3). After 2 and 3 weeks of ang II infusion, male rats had a significantly higher proteinuria than female rats. Furthermore, male rats had a significantly larger increase in proteinuria compared to baseline values than female rats (GEE analysis, interaction between ang II response and gender, *P* = 0.001). When corrected for body weight, proteinuria was still higher in males than in females (data not shown). Creatinine clearance corrected for body weight was not different between the four groups (ang II-treated rats: male, 8.9 ± 2.5 mL/min/kg, female, 8.3 ± 2.1 mL/min/kg; sham treated: male, 8.2 ± 1.9 mL/min/kg; female, 9.2 ± 1.9 mL/min/kg; *P* = 0.544, Kruskal–Wallis test) after 3 weeks of treatment.

#### Responses of intrarenal inflammatory parameters and kidney damage following ang II infusion

**Figure 4:**
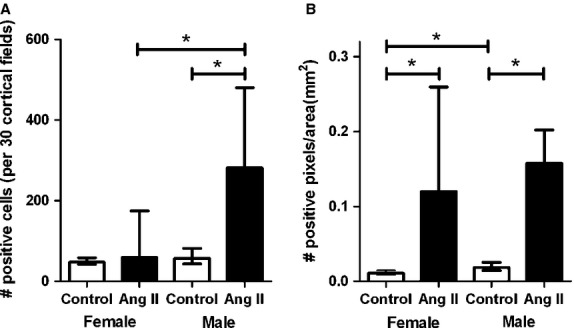
Median (interquartile range) numbers of ED-1-positive cells (A) and *α*-smooth muscle actin expression (B) in the interstitium of the kidney of female rats and male rats without chronic angiotensin II infusion (white bars) and with chronic angiotensin II infusion (black bars). Abbreviations: Ang II, angiotensin II. *P ≤ 0.05 (Mann–Whitney *U*-test).

In order to evaluate inflammation in the kidney, we studied the number of interstitial macrophages in the kidney sections (Fig.4A). The number of ED-1-positive macrophages per 30 cortical fields was not different between male and female sham-treated rats. After ang II treatment the number of interstitial macrophages was significantly increased as compared to sham-treated rats in males, but not in female rats. There were no significant differences in CD206-positive cells per 30 interstitial fields between sham-treated males and females and no effect of ang II infusion (data not shown).

In order to evaluate possible kidney damage, kidney sections were stained for *α*-SMA (Fig.4B). Figure4B shows that male sham-treated rats had significantly more expression of interstitial *α*-SMA than female sham-treated rats. In both male and female rats a significant increase in expression of interstitial *α*-SMA after ang II infusion was found as compared to the sham-treated groups. However, after ang II infusion no differences were found between female and male rats. There was a strong relationship between *α*SMA and blood pressure at 3 weeks (*R*^2^ = 0.282, *P* = 0.013).

#### AT_1_ and AT_2_-receptor mRNA

**Figure 5:**
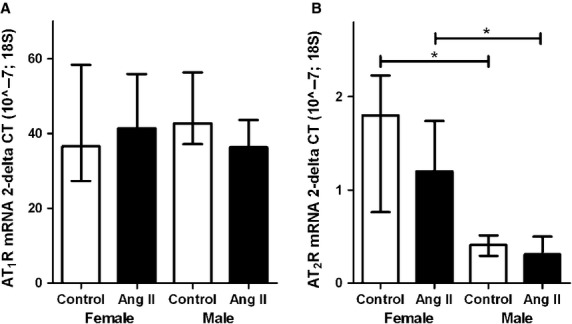
Median (with 25th and 75th percentile) angiotensin 1 receptor and angiotensin 2 receptor mRNA expression in the kidney (A and B respectively) of female and male rats without chronic angiotensin II infusion (white bars) and with chronic angiotensin II infusion (black bars). Abbreviations: ang II, angiotensin II; AT_1_-R, angiotensin II type 1 receptor; AT_2_-R, angiotensin II type 2 receptor. *P ≤ 0.05 (Mann–Whitney *U*-test).

AT_1_-R and AT_2_-R expression in kidney and aorta were evaluated (Fig.5A and B). No difference in mRNA expression for AT_1_-R in the kidney was found between sham-treated female and male rats and ang II infusion did not affect AT_1_-R expression in the kidney either. The mRNA expression for AT_2_-R in the kidney, however, was higher in female rats than in male rats, with again no effect of ang II treatment.

## Discussion

In this translational study we found differences in the response to ang II infusion in both healthy human subjects and in healthy rats. In humans, men showed an increased blood pressure and ERPF response, and a slightly decreased GFR response to acute ang II infusion compared with women. We showed that chronic ang II infusion resulted in a higher blood pressure in male compared with female rats, which confirms previous studies. However, we also showed an increased proteinuria response to chronic ang II infusion in male rats compared with female rats. This was previously only shown in spontaneous hypertensive rats (SHR; Sullivan et al., 2007). The increased ang II response in males was associated with more inflammation in the kidney compared with female rats. It may be speculated that our data point toward an increased ang II sensitivity in men, which may be involved in the higher risk for renal damage in males on the long term.

Our human data showed a higher blood pressure response to acute ang II infusion in men compared with women. These results differ from those from Miller et al. and Gandhi et al. (Gandhi et al. 1998; Miller et al. 1999), which might be due to differences in study protocol and patient selection. We feel that the main difference between our study and those of Miller and Gandhi is that we standardized measurements by phase of the menstrual cycle, as the latter can considerably affect RAAS-activity (Pechere-Bertschi and Burnier 2004). We found in both men and women the expected renal hemodynamic response to ang II infusion, namely a decrease in ERPF. This response is due to renal vasoconstriction, mainly in the efferent arteriole. However, the ERPF response is more pronounced in men compared with women. A greater decrease in ERPF in males may reflect a larger availability of the AT_1_-R, due to an upregulation of this receptor in a less active intrarenal RAAS system (Visser et al. 2008a,b). For GFR, the response was more pronounced in women than in men, since women responded to ang II by a significant decline in GFR, whereas men responded by maintaining GFR. This finding is consistent with the results of Miller et al. and it suggests that the afferent arteriole is more sensitive to ang II stimulation in women than in men. This different balance between afferent and efferent vasoconstriction in response to ang II in females compared to males could be a protective mechanism of the female glomerulus to prevent glomerular hypertension (Miller et al. 1999).

To evaluate the effect of chronic ang II infusion we studied the proteinuria and blood pressure response to 3 weeks of ang II infusion in male and female rats. In accordance with the acute effects of ang II on blood pressure in our human study and with results of other rat studies (Sampson et al. 2008; Tatchum-Talom et al. 2005; Xue et al., 2005; Ebrahimian et al. 2007), we observed a more rapid increase of blood pressure after ang II infusion in male compared with female rats. Furthermore, male rats also showed higher proteinuria at baseline and an increased proteinuria response after similar chronic ang II infusion compared with female rats. This finding suggests an increased sensitivity of the proteinuria response to ang II in males compared with females. To our knowledge, this is the first study showing this sex difference in proteinuria in response to chronic infusion of ang II in healthy rats. Sullivan et al. showed the same difference in SHR rats (Sullivan et al. 2007). Moreover, others have shown that transgenic rats bearing the mouse Ren2 gene have sex-dependent differences in proteinuria, with higher protein excretion in males compared to females (Pendergrass et al. 2008). This, however, appeared to be due to higher levels of ang II in male transgenic rats compared with female transgenic rats, rather than to higher ang II sensitivity. Our current data show that ang II-induced proteinuria after chronic ang II infusion in healthy rats is also sex dependent. The reason for the sex difference in proteinuria is not completely understood. Several mechanisms could be involved. It seems likely that hemodynamic effects are involved, since the ang II-induced blood pressure increase was more severe in males compared with females in our study. This may cause an increased pressure-induced injury in males compared with females (Remuzzi and Bertani 1998).

Inflammatory processes and/or fibrosis may also be involved in the sex differences in ang II-induced proteinuria (Noronha et al. 2002). Interstitial macrophage influx was different for males and females after 3 weeks of ang II infusion; we observed increased numbers of macrophages after 3 weeks of ang II infusion only in males. The increased numbers of interstitial macrophages could either be the cause or consequence of the increased protein excretion. Excessive tubular reabsorption of protein results in tubulo-interstitial infiltration of inflammatory cells, especially monocytes (Remuzzi and Bertani 1998). On the other hand, macrophages themselves have also been shown to induce kidney damage (Anders and Ryu 2011). Thus, our data suggest a possible ang II-induced vicious circle of renal damage which might be more prominent in males.

Despite the higher proteinuria in males there were little interstitial profibrotic changes in the kidney, as measured by *α*SMA. These changes were not different between males and females. In the present study, the subtle pro-fibrotic damage after 3 weeks of ang II infusion appeared to be blood pressure related since there was a strong relationship between *α*SMA and blood pressure. There was no difference in blood pressure after 3 weeks of ang II treatment between males and females, this may also explain why we did not detect a sex difference in profibrotic kidney damage. However, we cannot exclude that infusion of ang II of longer duration, or with higher doses would have result in larger sex differences in proteinuria and blood pressure and in sex differences in profibrotic markers. This should be subject of further research.

Differences in responsiveness to ang II between males and females could be due to difference in expression of the AT_1_-R and AT_2_-R and in the balance between the two. Although in the kidney the mRNA expression for the AT_1_-R was not different in males and females, AT_2_-R mRNA was higher in female rats compared with male rats. The increased expression of the AT_2_-R in females is in line with studies of the group of Denton et al., who suggested that the AT_2_-R plays a role in the decreased responsiveness to ang II of females (Sampson et al. 2008; Brown et al. 2012; Safari et al. 2012; Sampson et al. 2012; Hilliard et al. 2013). As pointed out we infused ang II for only 3 weeks. This resulted in relatively little profibrotic damage and proteinuria, while we did not observe renal damage (as characterized by glomerular sclerosis - data not shown). The question arises whether prolonged infusion or a combination of ang II infusion with another hit (e.g. nephrectomy) does induce differences in renal damage in male and female rats. Moreover, the present experiments (both human and rats) were performed in young adults. Therefore, the differences observed in the RAAS in the present study may not be relevant to older individuals, such as older men and postmenopausal women.

In conclusion, in both humans and rats, males seem to have a higher vascular and renal sensitivity to ang II compared to females. In response to acute ang II infusion, men have a stronger rise in blood pressure and a stronger ERPF response, but a decreased GFR response than women. In rats, pressor responses during chronic ang II infusion are also larger in males than in females, and associated with a higher increase of proteinuria and intrarenal inflammation in males. This study showed that differences in function of the RAAS between males and females, including differences in presence and function of the AT_2_-R, could be an explanation for the gender differences in development of renal disease. Further research is required to elucidate these gender differences in RAAS regulation, which might contribute to a better view on the differences in renal risk profile and treatment recommendations between men and women.
